# Effect of Oral Administration of Magnesium on Cisplatin-Induced Nephrotoxicity in Normal and Streptozocin-Induced Diabetic Rats

**DOI:** 10.5812/numonthly.11624

**Published:** 2013-08-20

**Authors:** Nepton Soltani, Mehdi Nematbakhsh, Fatemeh Eshraghi-Jazi, Ardeshir Talebi, Farzaneh Ashrafi

**Affiliations:** 1 Research Center for Molecular Medicine, Hormozgan University of Medical Sciences, Bandar Abbas, IR Iran; 2 Department of Physiology, Hormozgan University of Medical Sciences, Bandar Abbas, IR Iran; 3 Water and Electrolytes Research Center, Isfahan University of Medical Sciences, Isfahan, IR Iran; 4 Department of Physiology, Isfahan University of Medical Sciences, Isfahan, IR Iran; 5 Isfahan^MN^ Institute of Basic and Applied Sciences Research, Isfahan, IR Iran; 6 Department of Clinical Pathology, Isfahan University of Medical Sciences, Isfahan, IR Iran; 7 Department of Internal Medicine, Isfahan University of Medical Sciences, Isfahan, IR Iran

**Keywords:** Cisplatin, Streptozocin, Diabetes Mellitus, Magnesium, Dietary Supplements

## Abstract

**Background:**

Cisplatin (CP) therapy as the most common potent chemotherapeutic process is accompanied by nephrotoxicity. The diabetic state may protect rat kidney against this toxicity, and magnesium (Mg) on the other hand may reduce the glucose level in diabetic animals.

**Objectives:**

Current study was planned to investigate the effect of oral administration of magnesium supplementation on CP-induced nephrotoxicity in normal and Streptozocin (STZ)-induced diabetic rats.

**Materials and Methods:**

Male Wistar rats were divided into seven groups and underwent two experiment protocols. As protocol 1, group 1 was considered as the sham group. Group 2 (CP group) received CP (2 mg/kg/d) for five consecutive days. Group 3 (CP + Mg group) received magnesium sulphate (MgSO4, 10 g/L added to the drinking water) for 10 days and then treated with CP from sixth day. As protocol 2, animals received a single dose of STZ (65 mg/kg i.p.). Three days after diabetes induction, animals were divided into four groups; Groups 4 (D group), 5 (D + CP group), and 7 (D + Mg + CP group) followed the same manner as groups 1 to 3, respectively; and group 6 (D + Mg group) was treated with MgSO4 alone for 10 days. Finally, blood samples were obtained, and all animals were killed for kidney tissue investigation.

**Results:**

CP administration in normoglycemic rats significantly elevated the serum levels of blood urea nitrogen (BUN) and creatinine (Cr) (P < 0.05). However, coadministration of CP and Mg statistically increased the serum levels of BUN and Cr in both normoglycemic and diabetic animals when compared to the rats treated with CP alone (P < 0.05), while the serum level of Mg was significantly increased in nondiabetic groups (P < 0.05). No significant changes were observed in serum and kidney levels of nitrite; as well as the testis weight between all normoglycemic groups, whereas Mg decreased kidney levels of nitrite in diabetic groups when accompanied by CP (P < 0.05). The kidney and serum levels of malondialdehyde (MDA) enhanced significantly in nondiabetic rats treated with Mg and CP (P < 0.05). Kidney tissue damage score (KTDS), kidney weight, and body weight loss were significantly different among normoglycemic groups (P < 0.05), and Mg promoted the KTDS in diabetic animals treated with CP.

**Conclusions:**

Oral Mg supplementation did not protect the CP induced nephrotoxicity in diabetic rats.

## 1. Background

Cis-Diamminedichloroplatinum (II) (cisplatin; CP) as the most common potent chemotherapeutic drug is used in treatment of various solid tumors in clinic. The major side effect of CP is nephrotoxicity which is characterized by diminished renal blood flow and glomerular filtration rate, and is accompanied by increased blood urea nitrogen (BUN) and creatinine (Cr) (1-7) and decline in magnesium (Mg) levels (8, 9).

On the other hand, hypomagnesaemia is one of the complications of diabetes pathogenesis (10). Recent studies have shown that in diabetic rats, Mg deficiency is induced by Streptozocin (STZ) (11-15). Mg as an essential ion plays important roles in physiological processes. Administration of Mg supplementations improves endothelial function (16) and restores hemodynamic and tubular function in postischemic rats (17). Mg is also involved in glucose metabolism and insulin action (10). Administration of Mg supplementation reduces the oxidative stress in Alloxan-induced diabetic rats (18). Recent studies have shown that oral administration of Mg compounds may be able to attenuate hypomagnesaemia (11, 13, 15), hyperglycemia (13, 14), and thermal hyperalgesia (15) in diabetic rats; and also prevent vascular complication induced by the diabetic state (11). Studies have indicated that uncontrolled STZ-induced diabetes in rats could protect renal tissue against CP-induced damage (19-22). The effect of Mg supplementations on animal models of nephrotoxicity induced by nephrotoxins was also reported in others studies (23, 24). No protective effect was reported against CP-induced nephrotoxicity by Mg administration in rats (23).

## 2. Objectives

There is no evidence for the protective role of Mg in CP-induced nephrotoxicity in diabetic state. Accordingly, we attempted to investigate the effect of oral Mg supplementations in CP-induced nephrotoxicity in diabetic and normal rats.

## 3. Materials and Methods

### 3.1. Drugs

CP was purchased from EBEWE Pharma Ges.m.b.H (Austria), STZ and Mg sulfate (MgSo4) were purchased from Sigma-Aldrich (St. Louis MO, USA).

### 3.2. Animals

Fifty three male Wistar rats (weighting 164 ± 2.1 g) were used in this study. Animals were housed at a room temperature of 23-25 ºC and 12h light/12h dark cycle and free access to water and rat chow. All procedures of this research were approved by the criteria outlined in the guide for care and use of laboratory animals (NIH US publication 86-23 revised 1985).

### 3.3. Experimental Protocol

The animals were divided into seven experimental groups to undergo two experiment protocols.

Protocol 1: Effect of Mg on CP-induced nephrotoxicity.

Three groups of animal were served in protocol 1 as follows:

Group 1; sham group: No treatment was applied to this group during the experiment.

Group 2; CP group: The group received CP (2 mg/kg/d) for 5 days.

Group 3; Mg + CP group: The group was treated with MgSo4 (10 g/L) added to drinking water for 10 days, and from sixth day, the animals were treated with CP as group 2.

Protocol 2: Effect of Mg and STZ induced diabetes on CP-induced nephrotoxicity.

In this protocol, diabetes was induced in rats with a single intraperitoneal injection of STZ (65 mg/kg). Three days after STZ administration, nonfasting blood glucose level was determined using glucometer (Ascensia Elite XL) and the rats with blood glucose levels above 250 mg/dL were considered diabetic and were selected for the experiment. The diabetic rats were randomly divided into four groups as follows:

Group 4; D group: No treatment was applied to this diabetic group during the experiment.

Group 5: D + CP group: Similar to group 2, this group received CP (2 mg/kg/d) for 5 days.

Group 6: D + Mg group: The group was treated with MgSo4 added to drinking water for 10 days.

Group 7: D + Mg + CP group: The group was treated with MgSo4 similar to group 6, but from sixth day, the animals were treated with CP similar to group 5.

All animals were killed after blood sampling. The serum samples were collected and stored at -20 ºC until measurement. The kidney and testis were immediately removed and weighed. The kidney and testis weights (KW and TS) were normalized to the body weight, and reported as tissue weight (g)/100 g of body weight. The right kidney was fixed in 10% formalin for pathological investigations, and the left kidney was homogenated in 2 mL of saline, centrifuged at 15000 rpm for 2 min, and the supernatant was collected for measurements.

### 3.4. Measurements

The serum levels of Cr, BUN, and Mg were measured using diagnostic kits (Pars Azmoon Co., Tehran, Iran). The serum and renal levels of nitrite (NO stable metabolite) were determined by a commercial kit (Promega Corporation, Madison, WI, The USA). The renal and serum levels of malondialdehyde (MDA) were measured by manual method. Briefly, 0.5 mL of the sample was mixed with 1 mL of 10% Trichloroacetic acid (TCA). The mixture was centrifuged at 2000 g for 10 min. Then, 500 µL of the supernatant was added to 500 µL of 0.67% Thiobarbituric acid (TBA) and was incubated in boiling water for 10 min. After cooling down, the absorbance was read at the wavelength of 532 nm.

### 3.5. Histopathological Procedures

For histopathological investigations, the excised right kidney was embedded in paraffin. Then, Hematoxylin and Eosin stain was used to assay the tubular damage. The tubular damage was evaluated by an expert pathologist who was blinded to the study regarding tubular dilation and simplification, tubular cell swelling and necrosis, tubular casts and intra luminal cell debris with inflammatory cells infiltration. According to intensity of tubular injuries, the kidney tissue damage score (KTDS) was assigned in the range of 1 to 4; while zero score was assigned to normal tissue without any damage.

### 3.6. Statistical Analysis

Data was expressed as mean ± SEM. The comparison of body weights between the groups was performed using repeated measure analysis. The levels of BUN, Cr, Mg, MDA, nitrite, KW, and TS were analyzed by one-way ANOVA followed by the least significant difference (LSD) test. The groups were compared regarding KTDS using Kruskal-Wallis or Mann-Whitney tests.

## 4. Results

### 4.1. Effect of Mg on CP-Induced Nephrotoxicity

**Figure 1.**
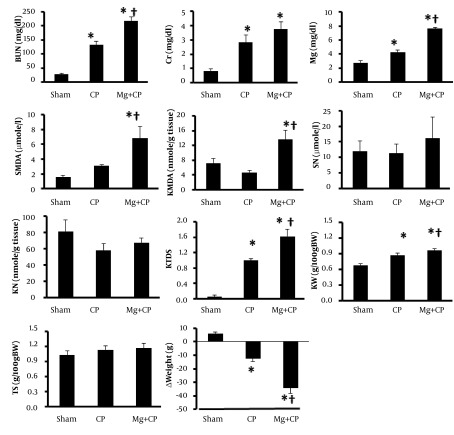
The Measured Biochemistry Parameters in Serum and Kidney, and Kidney Damage, Kidney Weight, Testis Weight and Body Weight in Four Experimental Groups. The data is reported as mean ± SEM. * and † indicate significant difference from the Sham and CP groups, respectively (P < 0.05). BUN: blood urea nitrogen, Cr: creatinine, SMDA: serum malondialdehyde, Mg: magnesium, SN: serum nitrite, KMDA: kidney MDA, KN: kidney nitrite, KTDS: kidney tissue damage score, KW: kidney weight per 100 g body weight, TS: testis weight per 100 g body weight, ∆Weight: body weight change, D: diabetic rats, D + CP: diabetic rats treated with cisplatin (CP), D + Mg: diabetic rats treated with magnesium (Mg), and D + Mg + CP: diabetic rats treated with the combination of Mg and CP.

Administration of CP was accompanied with significant increase in serum levels of BUN and Cr, and increase of KW and KTDS, which revealed induced nephrotoxicity (P < 0.05) (Figure 1). A significant weight loss was also observed with CP (P < 0.05). No significant changes were found by CP alone in the serum and kidney tissue levels of MDA and nitrite, and TS. Administration of Mg accompanied with CP changed the serum levels of BUN, Cr, Mg, and MDA, and kidney tissue MDA, KW, KTDS, and weight loss toward a significant larger value (Figure 1), which revealed no-protective role of oral administration of Mg on CP-induced nephrotoxicity.

### 4.2. Effect of Mg and STZ-Induced Diabetes on CP-Induced Nephrotoxicity

**Figure 2.**
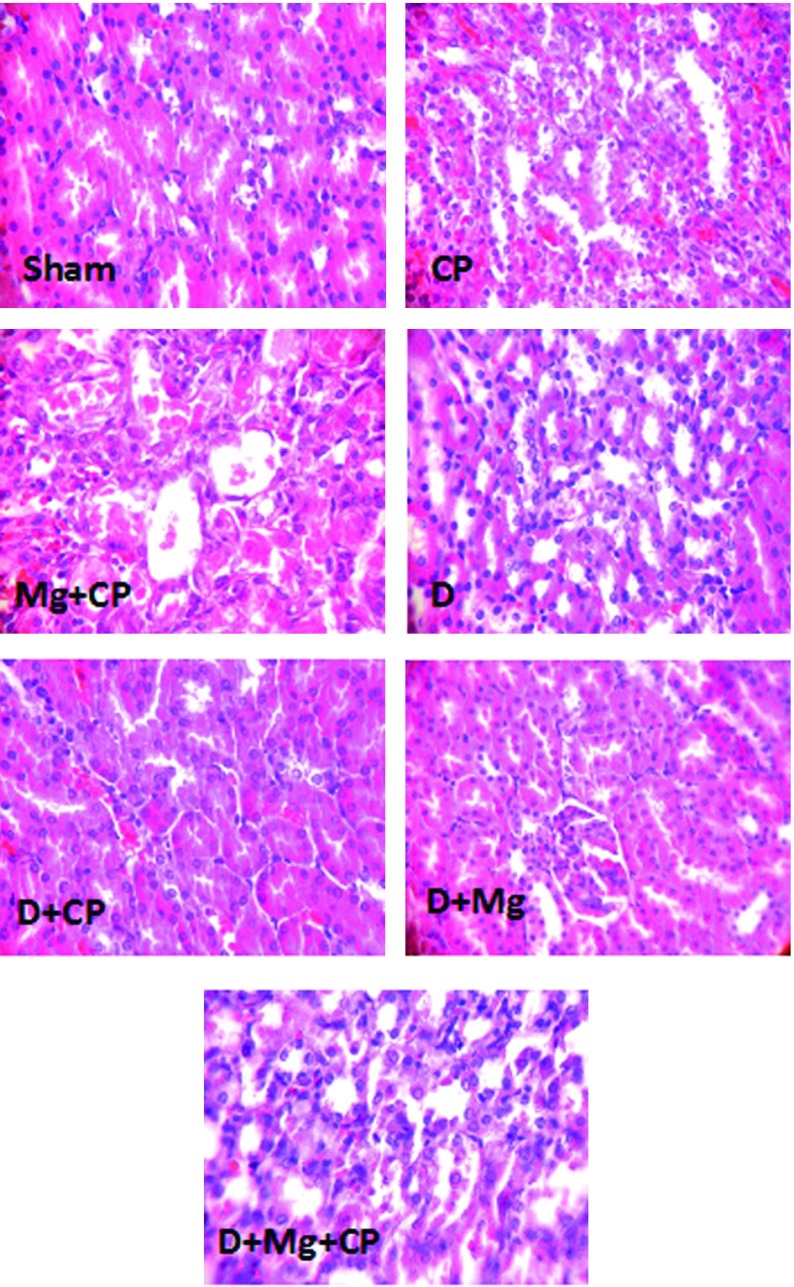
The Images of Kidney Tissues (Magnification x 100) in Sham, Cisplatin (CP) Alone Treated, Magnesium Plus CP (Mg + CP) Treated, Diabetic rats (D), Diabetic rats Treated With CP (D + CP), Diabetic Rats Treated With Mg (D + Mg), and Diabetic Rats Treated With the Combination of Mg and CP (D + Mg + CP). More Kidney Tissue Damage Was Observed in Mg + CP and D + Mg + CP Groups, While no Damage Was Detected in the Sham and D Groups.

CP did not increase the serum levels of BUN and Cr in diabetic rats when compared to the diabetic group not treated with CP. In diabetic rats, when Mg was accompanied with CP, the serum levels of BUN (significantly, P < 0.05) and Cr (non-significantly) increased. The KTDS in diabetic rats treated with the combination of CP and Mg (group 7) increased significantly when compared to other groups (P < 0.05). However, no significant differences in the serum levels of MDA, Mg, and nitrite were detected between the groups. No difference was also obtained in kidney tissue level of MDA between the groups. However, Mg significantly decreased the kidney tissue level of nitrite (P < 0.05). The weight loss in CP treated groups (groups 5 and 7) was significantly greater than those in other groups not treated with CP (P < 0.05) (Table 1). The images of kidney tissue are demonstrated in Figure 2.

**Table 1.** The Measured Biochemistry Parameters in Serum and Kidney, and Kidney Damage, Kidney Weight, Testis Weight and Body Weight in Four Experimental Groups. The data is reported as mean ± SEM.

Group	BUN^a^ (mg/dL)	Cr^a^ (mg/dL)	SMDA^a^ (µmole/L)	KMDA^a^ (nmole/g tissue)	Mg^a^ (mg/dL)	SN^a^ (µmole/L)	KN^a^ (nmole/g tissue)	KTDS^a^	KW^a^ (g/100gBW)	TS^a^ (g/100gBW)	∆Weight^a^ (g)
**D ** ^**b**^	49.6 ± 4.3	0.87 ± 0.11	7.4 ± 1.4	4.7 ± 0.8	4.4 ± 0.5	16.4 ± 5.3	70.4 ± 9.8	0.2 ± 0.1	1.00 ± 0.03	1.10 ± 0.05	-8.1 ± 1.78
**D + CP** ^**c**^	50.5 ± 8.5	0.93 ± 0.17	6.3 ± 1.6	5.6 ± 1.4	3.6 ± 0.5	10.7 ± 2.4	79.4 ± 11.4	0.3 ± 0.2	1.04 ± 0.05	1.17 ± 0.12	-18.1 ± 3.15^h,i^
**D + Mg** ^**d**^	42.4 ± 3.1	1.01 ± 0.18	7.2 ± 2.1	5.5 ± 1.4	3.2 ± 0.4	17.2 ± 7.3	42.4 ± 3.1^g,h^	0.2 ± 0.2	0.96 ± 0.03	1.22 ± 0.11	-1.0 ± 4.81
**D + Mg + CP ** ^**e**^	99.3 ± 11.1^f^	1.20 ± 0.42	5.4 ± 1.4	6.0 ± 1.5	4.8 ± 0.8	23.1 ± 7.7	51.4 ± 8.3^h^	0.7 ± 0.2^f^	0.98 ± 0.04	1.07 ± 0.09	-19.6 ± 2.14^h,i^

^a^Abbreviations: BUN; blood urea nitrogen, Cr; creatinine, KMDA; kidney MDA, KN; kidney nitrite, KTDS; kidney tissue damage score, KW; kidney weight, Mg; magnesium, SN; serum nitrite, SMDA; serum malondialdehyde, TS; testis weight, ΔWeight; body weight change

^b^D; diabetic rats

^c^D + CP: diabetic rats treated with Cisplatin (CP)

^d^D + Mg: diabetic rats treated with magnesium (Mg)

^e^D + Mg + CP: diabetic rats treated with the combination of Mg and CP

^f^Indicates significant difference from others groups (P < 0.05).

^g^Indicate significant difference from D + CP group

^h^Indicate significant difference from D group

^i^Indicate significant difference from D + Mg group (P < 0.05).

## 5. Discussion

The main objective of this study was to investigate the effect of Mg supplementation on CP-induced nephrotoxicity in normal and diabetic rats. Our results showed that administration of CP to normoglycemic rats induces an increase in the level of markers of renal function such as BUN and Cr. This was confirmed by data of pathology and KW. In contrast, administration of CP to diabetic rats had no effect on serum levels of BUN and Cr, and also no change was observed in KW and KTDS. It is documented that CP increases the serum levels of BUN and Cr in normal rats (1, 25) by oxidative and inflammatory processes (7, 26-28). Frequent evidence has indicated that diabetic rat kidney resists CP-induced nephrotoxicity (19-22) which may not be related to polyuria induced by diabetic state (22). Experimental data showed that renal CP accumulation in diabetic rats decreased after CP administration and also urinary CP excretion increased (19, 21). Valentovic et al. suggested that impairment in cellular uptake process can be associated with transport of CP (21). CP is carried into the proximal tubular cells by the organic cation transporter (OCT2) at the basolateral membrane (29, 30). This system is damaged in kidney of STZ-diabetic rats (31). Therefore, inefficacy of cellular transporter system in diabetic rats would lead to reduction of CP accumulation as well as increased excretion of CP. Mg accompanied with CP had no positive effects on CP-induced nephrotoxicity in both protocols; specified by an elevation in markers of renal function. Our results were in agreement with our recent study (23). CP-induced body weight loss may be associated with gastrointestinal disturbances (32, 33). Administration of Mg intensified weight loss induced by CP. Mg itself participates in gastrointestinal disorders (24, 34-36). Unfavorable effect of Mg and a nephrotoxin coadministration on weight loss was observed in previous investigations (23, 24). Another study demonstrated that gastrointestinal symptoms are common side effects of Mg supplementation in diabetic patients (37). On the other hand, hypomagnesaemia is one of the side effects of CP (38) which may happen two weeks after the administration of CP (39). Mg deficiency after administration of CP did not occur in normoglycemic CP-treated group owing to the duration of our experiment. Although, in previous studies (11-13) we observed plasma Mg depletion after diabetes induction, in the present study serum Mg level did not change in STZ-induced diabetic animals due to the duration of study. Also, serum level of Mg did not change in all Mg-treated diabetic groups. This finding is in agreement with our previous studies (11, 12) and intracellular magnesium shift may explain this finding (13). Mg accompanied by CP treatment in nondiabetic state increased kidney and serum levels of MDA in the present study. MDA as the final product of lipid peroxidation is one of the biomarkers of oxidative stress (40). In our study, administration of Mg accompanied by CP enhanced lipid peroxidation, which resulted in elevated values of MDA in the nondiabetic group, although the diabetic groups were not significantly different in this regard. In the present study, we showed that oral Mg administration in diabetic groups decreased renal level of NO consistently, Nagaei et al. demonstrated that administration of water containing Mg ion prevents side effects of Loxoprofen in adjuvant-induced arthritis rats by decreasing iNOS and NO levels in gastric mucosa (41). In another investigation, Mg treatment elevated renal content of NO in nephrotoxicity model induced by cyclosporine (24). Evidence has shown that NO agent acts in various manners against CP-induced nephrotoxicity (42, 43). Thus, the mechanism involved is not well elucidated. Here, a problem about unfavorable effect of Mg administration on CP-induced nephrotoxicity in both normal and diabetic rats is proposed. There is a correlation between Mg and CP. The evidence available demonstrated that OCT2 system is up-regulated in hypomagnesaemia state, which raises CP accumulation in renal tissue (44, 45). In our recent study, low dose Mg supplementation exacerbated side effects of CP administration (23) due to possible above mentioned reason, which is in agreement with the results obtained in the current study.

Conclusion: It is concluded that diabetic state protects renal tissue against nephrotoxicity induced by CP. Magnesium supplementation intensifies CP-induced injury in normal and diabetes animal models.
